# Post-COVID sequelae effect in chronic fatigue syndrome: SARS-CoV-2 triggers latent adenovirus in the oral mucosa

**DOI:** 10.3389/fmed.2023.1208181

**Published:** 2023-06-29

**Authors:** Ulf Hannestad, Eirini Apostolou, Per Sjögren, Björn Bragée, Olli Polo, Bo Christer Bertilson, Anders Rosén

**Affiliations:** ^1^Department of Biomedicine and Surgery, Division of Cell Biology, Linköping University, Linköping, Sweden; ^2^Department of Neurobiology, Care Sciences and Society, Division of Family Medicine and Primary Care, Karolinska Institute, Stockholm, Sweden; ^3^ME-Center, Bragée Clinics, Stockholm, Sweden

**Keywords:** post-COVID condition, SARS-CoV-2, myalgic encephalomyelitis/chronic fatigue syndrome, ME/CFS, human adenovirus (HAdV), saliva antibodies, oral mucosa immune response

## Abstract

The post-viral fatigue syndromes long COVID and myalgic encephalomyelitis/chronic fatigue syndrome (ME/CFS) have multiple, potentially overlapping, pathological processes. These include persisting reservoirs of virus, e.g., SARS-CoV-2 in long COVID patient’s tissues, immune dysregulation with or without reactivation of underlying pathogens, such as Epstein–Barr virus (EBV) and human herpesvirus 6 (HHV6), as we recently described in ME/CFS, and possibly yet unidentified viruses. In the present study we tested saliva samples from two cohorts for IgG against human adenovirus (HAdV): patients with ME/CFS (*n* = 84) and healthy controls (*n* = 94), with either mild/asymptomatic SARS-CoV-2 infection or no infection. A significantly elevated anti-HAdV IgG response after SARS-CoV-2 infection was detected exclusively in the patient cohort. Longitudinal/time analysis, before and after COVID-19, in the very same individuals confirmed HAdV IgG elevation after. In plasma there was no HAdV IgG elevation. We conclude that COVID-19 triggered reactivation of dormant HAdV in the oral mucosa of chronic fatigue patients indicating an exhausted dysfunctional antiviral immune response in ME/CFS, allowing reactivation of adenovirus upon stress encounter such as COVID-19. These novel findings should be considered in clinical practice for identification of patients that may benefit from therapy that targets HAdV as well.

## Introduction

There is abundant evidence for infection as a trigger of chronic fatigue, e.g., EBV-driven infectious mononucleosis (IM) causing post-viral fatigue, *Coxiella burnetii* initiating post-Q fever fatigue, Ebola virus activating post-Ebola fatigue, SARS-corona virus from 2003 inducing post-SARS syndrome, and now recently SARS-CoV-2 triggering long COVID (1, 2). These post-viral fatigue syndromes are debilitating illnesses affecting millions of individuals worldwide. The pathophysiological mechanisms are complex (1) and involve autoimmune and dysfunctional antiviral and metabolic mechanisms including failure of aerobic energy production (3, 4). The symptoms have been studied extensively in ME/CFS and include post-exertional malaise (PEM)—the cardinal diagnostic symptom, postural tachycardia syndrome (POTS), myalgia, dysautonomia with unrestored sleep, and neurocognitive disturbance (brain fog) (5). Risk factors for developing long COVID include type 2 diabetes, SARS-CoV-2 RNAemia, EBV-viremia, and certain autoantibodies (6). ME/CFS is often (up to 70% of cases as reported in some studies) triggered by EBV-induced IM (2), and associates also with reactivation of herpesviruses, i.e., EBV and human HHV6 (1, 2). However, no convincing data are available to assign a defined causative pathogen for ME/CFS, albeit human herpesviruses and enteroviruses have been indicated (7). Previous studies have also investigated whether ME/CFS is associated with human adenovirus (HAdV) by employing antibody serology-analysis of plasma samples. No association was found, however (8, 9).

Here, we have explored HAdV reactivation in the oral mucosa and have extended our recent study by Apostolou et al. (10), in which we found strong reactivation of *Herpesviridae* family members, e.g., EBV, HHV6, as well as human endogenous retrovirus K (HERV-K) in saliva after mild/asymptomatic SARS-CoV-2 infection. HAdV are pathogenic DNA viruses both in humans and animals. There are over 110 HAdV types based on their unique genomic characteristics, which are classified into seven, serologically different, species (A–G) (11). After initial infection HAdV can establish a persistent, latent infection in various cell types. Typical sites are adenoids, tonsils, and gut-associated lymphoid tissue (12). Occasionally HAdV infections can cause keratoconjunctivitis, haemorrhagic cystitis, hepatitis, haemorrhagic colitis, pancreatitis, nephritis, or meningoencephalitis either after primary infection or after reactivation (13). In this study we have investigated the presence of HAdV by analyzing anti-HAdV antibody “fingerprints,” based on the fact that antiviral IgG is detectable in a wider time-window (months) compared to virus detection by polymerase chain reaction (PCR), where viral DNA can be detected in a narrow time interval (days) after acute infection.

## Methods

### Human saliva panel

Saliva samples were collected from 84 ME/CFS patients that were recruited to this study from the Bragée Clinic, Stockholm, Sweden. All patients were diagnosed with ME/CFS according to the 2003 Canadian Consensus Criteria before the COVID-19 pandemic (3). Saliva samples were also collected from 94 healthy control donors (HD) recruited from Linköping University and Hospital. None of the participants were vaccinated against SARS-CoV-2 at the start of the study (inclusions from June till December 2020). Description of saliva collection, handling of samples, study participant enrolment is detailed in Apostolou et al. (10). The study was reviewed and approved by the Swedish Ethical Review Authority, Regional Ethics Committee (D.nr. 2019-0618).

### Analysis of virus IgG in oral mucosa

Anti-HAdV IgG antibodies in saliva were used as markers for HAdV reactivation. The specific adenovirus IgG was analyzed in saliva samples (diluted 1:4), and in plasma samples (diluted 1:100), using an anti-HAdV IgG ELISA kit from EUROIMMUN AG (Lübeck, Germany) according to manufacturer’s instructions. Antibodies against SARS-CoV-2 receptor-binding domain of the spike protein (RBD), and anti-nucleoprotein (NP) antibodies were tested in multiplex assay as previously described (10).

### Statistics

Data were analyzed for the determination of statistical significance of the observed differences between groups, with a *p-*value < 0.05 considered as significant. All statistical analyses were performed using the SAS Institute JMP program (v 13.2.1) or GraphPad Prism software (v.9.1.2).

For the comparisons between ME/CFS and HDs groups, we used the non-parametric Kruskal-Wallis test for multiple comparisons, since data was not normally distributed, and controlled for false discovery rate (5%) by using two-stage Benjamini, Krieger and Yekutieli (BKY) procedure (14). The data from plasma samples were normally distributed and hence one-way ANOVA was used for multiple comparisons. Multiple linear regression was performed for the determination of confounding factors (age, sex, mononucleosis) and controlled for false discovery rate of 5% according to BKY. Statistically significant differences are indicated in the figures as *p < 0.05, **p < 0.01. Non-parametric bi-variate analysis was performed according to Pearson, e.g., linear regression, in order to analyze possible correlation between anti-HAdV IgG in plasma and anti-HAdV IgG in saliva.

## Results

Saliva samples were collected from patients with ME/CFS (n = 84) and healthy control donors (HD; *n* = 94) and analyzed for anti-HAdV IgG and anti-SARS-CoV-2 IgG as described in Methods. Both patients and controls were divided in three groups based on their immune response to SARS-CoV-2: 1. Participants with a systemic response (IgG in plasma, with or without saliva IgG), termed systemic-ME or systemic-HD; 2. Participants with local (saliva) response only, named local-ME or local-HD; 3. participants with no anti-SARS-CoV-2 response (negative-ME or negative-HD).

**Figure 1:**
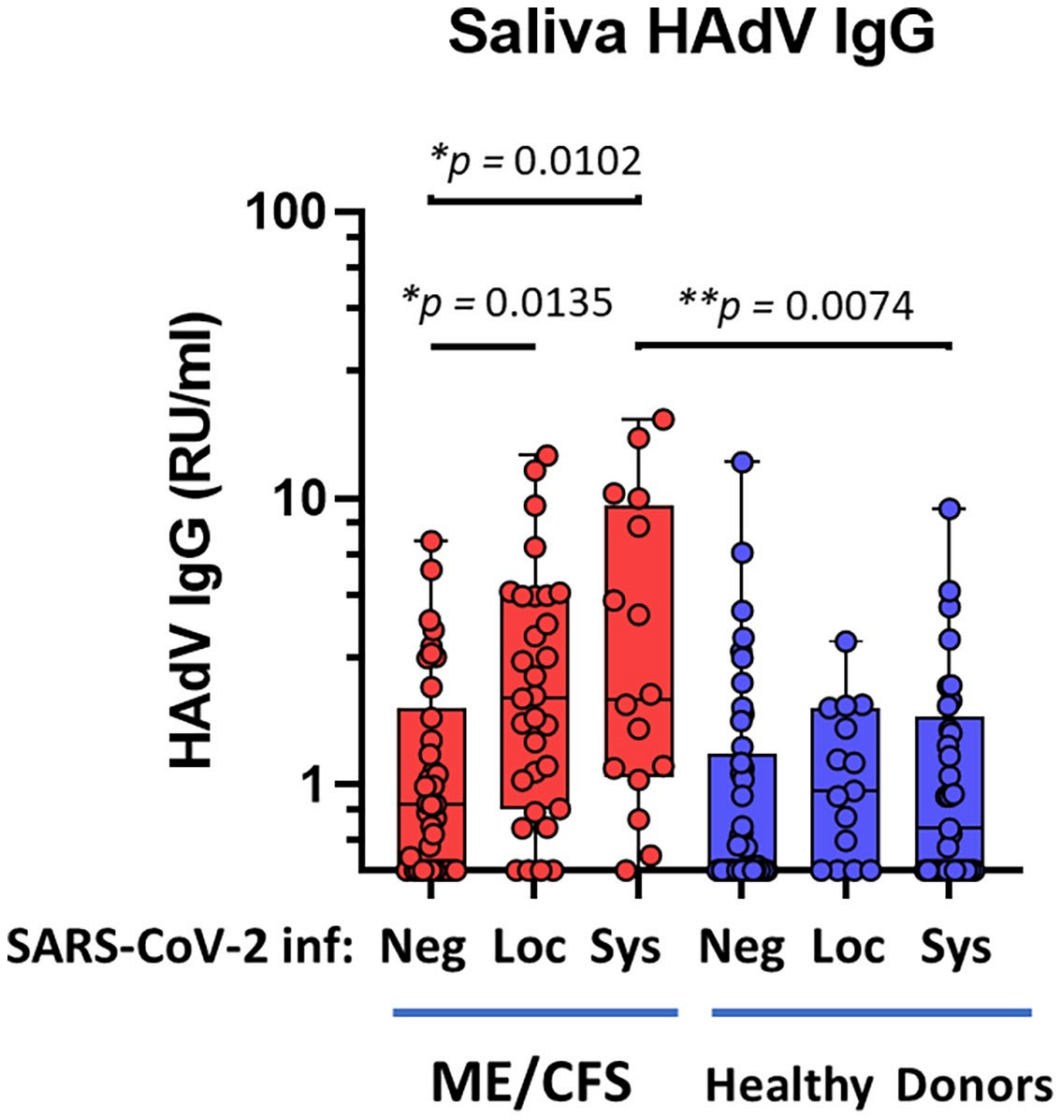
Saliva antibody reactivity to human adenoviruses in patients with ME/CFS (*n* = 84, red dots/boxes) and healthy donors (*n* = 94, blue dots/boxes). Sys: participants anti-SARS-CoV-2 RBD (spike protein receptor binding domain)-positive for systemic response in plasma (with or without saliva IgG; ME, *n* = 18; HD, *n* = 33). Loc, participants RBD-positive for local response in saliva (ME, *n* = 31; HD, *n* = 15). Neg, participants RBD IgG-negative both in plasma and saliva (ME, *n* = 38; HD, *n* = 46). Data are presented as boxplots with median values with minimum to maximum whiskers. Statistically significant differences according to non-parametric Kruskal-Wallis procedure and false discovery rate adjustment (5%) according to Benjamini, Krieger and Yekutieli procedure (14) are indicated as *p < 0.05, **p < 0.01. RU/ml, relative units per milliliter.

Our data reveals a significantly elevated IgG response in saliva against HAdV following SARS-CoV-2 infection in patients with ME/CFS, whereas no difference was found in HDs (Figure 1): negative-ME vs. systemic-ME (*p = 0.0102), and negative-ME vs. local-ME (*p = 0.0135); negative-HD vs. systemic-HD (*ns*, *p* = 0.3479), and negative-HD vs. local-HD (*ns, p* = 0.1796). More importantly, comparing the HD cohort to the ME/CFS cohort, significantly elevated HAdV IgG levels were found in patients with ME/CFS with a systemic SARS-CoV-2-response compared to HD with a systemic SARS-CoV-2 response (**p = 0.0074; Figure 1).

Baseline antibody responses against HAdV in the local oral mucosa, independently of SARS-CoV-2 infection, e.g., negative-ME vs. negative-HD cohorts, were not significantly different (Figure 1).

We also tested whether gender, age, or a history of mononucleosis were confounding factors for anti-HAdV antibody levels. Multiple regression analysis was performed using Benjamini, Hochberg, Yekutieli FDR of 5% (14). Gender, age, or mononucleosis were found not to be confounding factors for saliva HAdV IgG levels.

**Figure 2:**
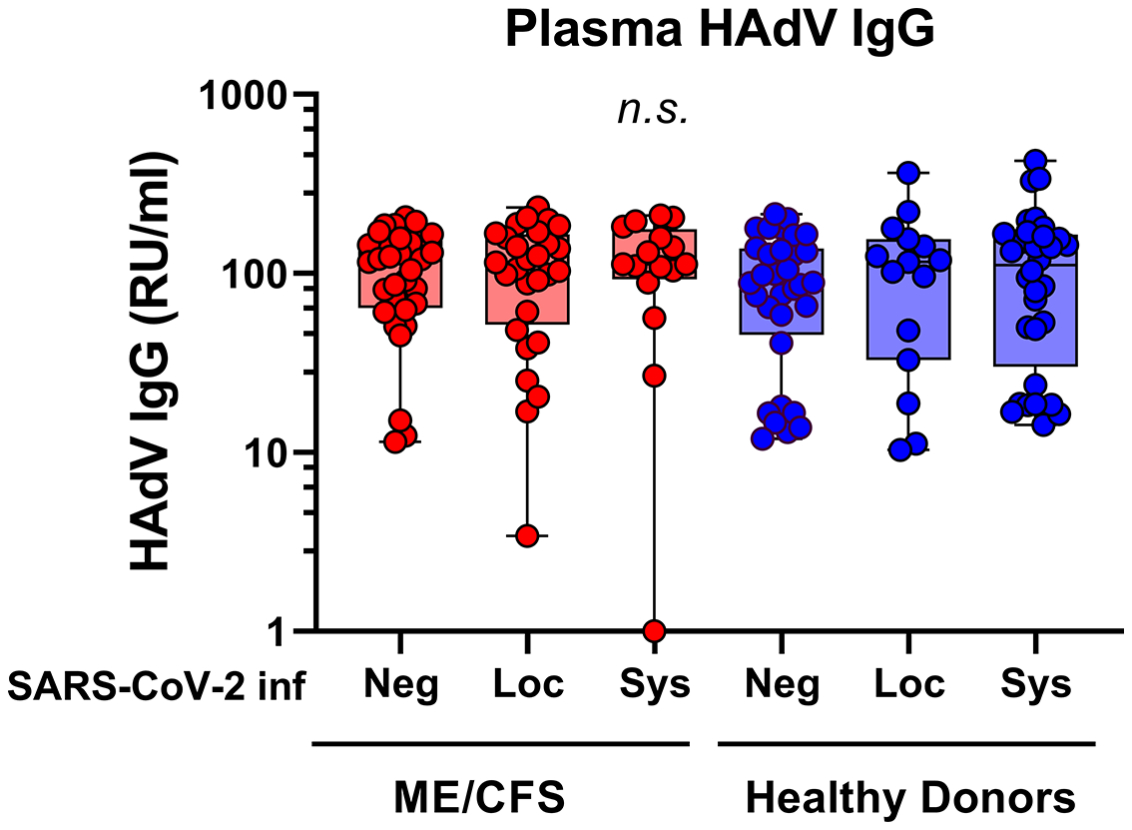
Plasma antibody reactivity to human adenoviruses in patients with ME/CFS (*n* = 75, red dots/boxes) and healthy donors (*n* = 79, blue dots/boxes). Sys: participants SARS-CoV-2 RBD (RBD)-positive for systemic response in plasma (with or without saliva IgG; ME, *n* = 15; HD, *n* = 32). Loc, participants RBD-positive for local response in saliva (ME, *n* = 28; HD, *n* = 15). Neg, participants RBD IgG-negative both in plasma and saliva (ME, *n* = 32; HD, *n* = 32). Data are presented as boxplots with median values with minimum to maximum whiskers. Statistically significant differences were not found (*n.s*., non-significant) according to non-parametric Kruskal-Wallis procedure and false discovery rate adjustment (5%) according to Benjamini, Krieger and Yekutieli procedure (14). RU/ml, relative units per milliliter.

SARS-CoV-2 infection generates a distinct antibody fingerprint of latent HAdV reactivation in saliva, but not in plasma. The plasma anti-HAdV IgG showed no significant differences between the subgroups (Figure 2). Furthermore, there was no correlation between anti-HAdV IgG in plasma and anti-HAdV IgG in saliva (Pearson’s linear regression analysis, data not shown), indicating that the immune response in the local mucosal compartment is discrete from systemic immune response.

**Figure 3:**
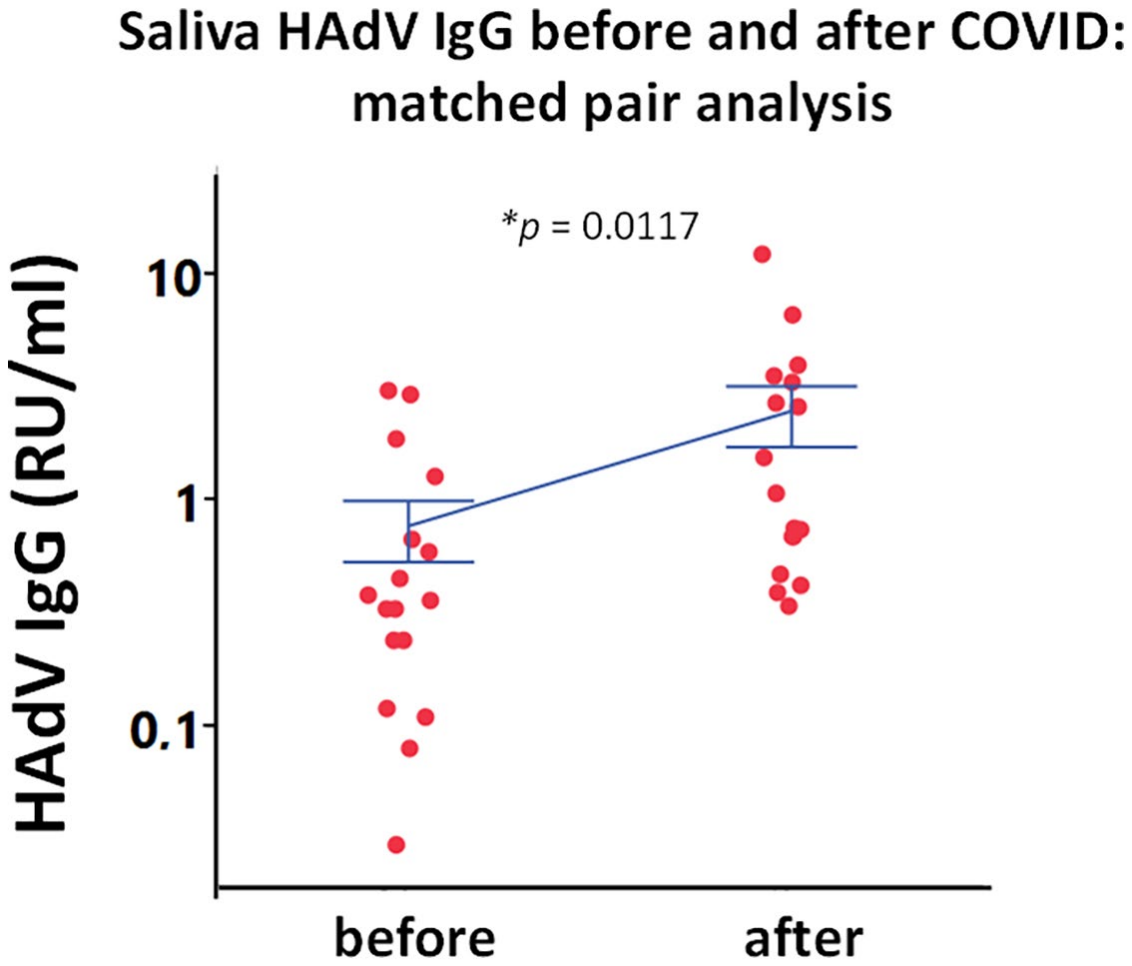
Paired analysis of saliva HAdV IgG from the same subjects before and after SARS-CoV-2 infection (*n* = 18). Individual points represent saliva HAdV IgG concentration as relative units per ml (RU/ml) in a longitudinal analysis of 18 individuals. Blue lines represent mean ± SEM. Statistically significant difference was tested in using matched pairs analysis platform (JMP statistical program). *p < 0.05 indicates statistically significant differences.

In order to validate the saliva HAdV IgG data, we performed a longitudinal analysis. Local reactivation of latent HAdV was confirmed in pairwise analysis by following individuals before and after SARS-CoV-2 infection. Matched pairs of saliva samples were analyzed in a subgroup of participants (*n* = 13 HDs, and 5 ME/CFS), who were infected with SARS-CoV-2 after the first round of sampling and during the course of this study (in the second pandemic wave between December 2020 to January 2021). Infection was documented by either PCR and/or established specific symptoms and was confirmed by the significant upregulation in RBD IgG (data not shown*, p = 0.03*) and IgM response (data not shown*, p* = 0.04). We found significant upregulation of HAdV IgG, within the same individuals when comparing antibody levels before and after SARS-CoV-2 infection using matched pair statistical analysis (Figure 3).

## Discussion

To the best of our knowledge this is the first report on adenovirus (re)activation after an infectious stress event—in this case SARS-CoV-2 infection, which is evident in saliva of patients with ME/CFS. Remarkably, the response was not observed in saliva from HD. The antibody levels were more pronounced in the subgroup of ME/CFS patients showing a systemic immune response to SARS-CoV-2 but was also significant in ME/CFS patients with a local SARS-CoV-2 response.

The results were validated in a longitudinal analysis of HAdV IgG level before and after SARS-CoV-2 infection during the first year of the pandemic. Data clearly indicate that SARS-CoV-2 reactivates latent HAdV in oral mucosa/tonsil tissue as determined by specific mucosal antibody “fingerprints.” The higher levels of antibody responses against HAdV in saliva that are indicative of HAdV reactivation were not detected in plasma. The lack of significant difference in the plasma in group comparisons, contrary to the strong statistical differences in saliva, was reported in our previous study for other latent viruses like EBV and HHV6 in the same cohorts (10). Adenoviruses rely in a latent state in the adenoids and tonsils of the oral cavity (11); therefore, their reactivation and subsequent immune responses are probably confined locally in saliva and hence easier to detect.

The antibody fingerprint of HAdV after mild/asymptomatic COVID-19 observed in the current study extends our previous data in which elevated saliva IgG against EBV, HHV6, and HERV-K was noted in ME/CFS, and in particular anti-EBV nuclear antigen 1 (EBNA1) IgG elevation was found to be exclusive for ME/CFS (10). The scenario is reminiscent of a chain-reaction, where one virus triggers a second virus, as exemplified by SARS-CoV-2 triggering EBV (10), and human endogenous retroviruses (HERVs). Activation of HERV by EBV might be the missing link between an initial EBV infection and the later onset of multiple sclerosis (MS) (15). There are considerable phenotypic and neuroimmune elements overlapping in ME/CFS and MS, including anti-EBNA1 IgG elevation (10, 16–18).

So, what is the cellular mechanism behind this HAdV elevated response effect? And why is it so unique for patients with ME/CFS and most likely patients with long COVID, as recently discussed by Davis et al. (1). Several studies report immunosuppressive effects exerted by a SARS-CoV-2 infection such as degradation of mitochondrial antiviral signaling protein (MAVS) and inhibition of type I interferon (IFN-I); reactivation of HHV6; HLA-G induced immunosuppression; reduced innate immunity, reduced antigen processing, reduced T-cell response (19–22). However, those patients had severe/moderate COVID-19 and were mainly hospitalized. In contrast, our study is focused on participants with mild/asymptomatic COVID-19. The fact that adenovirus was not activated in the oral mucosa of healthy donors, but only in patients with ME/CFS points toward a dysfunctional immune surveillance including exhausted antiviral interferon response after or even before infection by SARS-CoV-2, possibly due to previous encounter with HAdV and EBV that antagonize a proper DNA-sensing antiviral response (23, 24).

HAdV is ubiquitous in humans with nearly all individuals infected with at least one type by 6 years of age. Globally, a majority of adults have positive anti-HAdV IgG in plasma. HAdV can establish persistent infections with a chronic low-grade replication in different tissues, foremost tonsils and adenoids, and in the gastrointestinal tract gut-associated lymphoid tissue (11). Of particular importance is the fact that a majority of ME/CFS patients report irritable bowel syndrome with gut-symptoms like stomach cramps, bloating, diarrhea, and constipation, years prior to the onset of ME/CFS. This pre-ME/CFS stage could possible undermine a functional response to EBV-induced IM, which is a common trigger for ME/CFS disease onset (2). Furthermore, HAdV can be activated by a variety of stressors such as infections and toxins that exert a suppressive effect on the immune system (11).

Our findings also raise the question whether HAdV acts as a driver of the ME/CFS pathology, possibly in synergy with herpesviruses EBV and HHV6. Hanson and co-workers (9) recently published an extensive study including 122 different pathogens, i.e., HAdV, and plasma antibody response against these in ME/CFS, albeit their study did not implicate any one of the analyzed pathogens in ME/CFS, they write: “The possibility remains that ME/CFS cases arise from an uncommon variant of one or more enteroviruses or another type of virus and/or an uncommon reaction to a common endemic virus.” Here in our present study, we have detected a strong candidate for this common endemic virus: human adenovirus. We propose that HAdV acts as a chronic mucosal “irritant” that facilitates frequent reactivation of another common virus, the EBV—a proposal based on data presented here that HAdV is reactivated in ME/CFS, but not in HD, taken together with our recent data on mucosal elevation of EBV, HHV6 and HERV-K in ME/CFS (10). Clinically, our findings on HAdV potential involvement along with reactivated herpesviruses in the pathogenesis of ME/CFS and/or long COVID also open doors for novel antiviral therapy strategies. In clinical practice, particularly in immunosuppressed transplant patients, the antiviral brincidofovir (BCV) is being used to curb HAdV infections. BCV is a lipid conjugate of cidofovir that exerts high activity against double-stranded DNA viruses, especially adenovirus (25). In addition to antivirals, immune therapy is being explored for HAdV infection (11).

### Summary

We found a significantly elevated anti-adenovirus IgG titer in saliva of patients with ME/CFS after SARS-CoV-2 infection. This was not observed in healthy donors. We propose that this indicates an exhausted dysfunctional antiviral immune response in ME/CFS, allowing reactivation of adenovirus upon stress encounter such as COVID-19. Our findings demand further large-scale studies to better understand the role of HAdV both in ME/CFS and long COVID.

## Data availability statement

The original contributions presented in the study are included in the article/supplementary material, further inquiries can be directed to the corresponding author.

## Ethics statement

The study was reviewed and approved by the Swedish Ethical Review Authority, Regional Ethics Committee (D.nr. 2019-0618). The patients/participants provided their written informed consent to participate in this study.

## Author contributions

UH and AR conceptualized and conducted experiments, analyzed data, and wrote the manuscript. EA collected samples, organized the biobank, analyzed data, and wrote the manuscript. PS, BBe, OP, and BBr were responsible for patient contacts and provided samples and clinical data. AR supervised and funded the project. All authors contributed to the article and approved the submitted version.

## Funding

This study was supported by Swedish Research Council (4.3-2019-00201 GD-2020-138), Swedish Cancer Society (no. 211832Pj01H2/Infection-Autoimmunity-B-lymphoma grant to AR), and local Linköping University funds (AR).

## Conflict of interest

PS, BBe, BBr, and OP declare disclosure of interest as having income from Bragée Clinics and BBr being a partial owner.

The remaining authors declare that the research was conducted in the absence of any commercial or financial relationships that could be construed as a potential conflict of interest.

## Publisher’s note

All claims expressed in this article are solely those of the authors and do not necessarily represent those of their affiliated organizations, or those of the publisher, the editors and the reviewers. Any product that may be evaluated in this article, or claim that may be made by its manufacturer, is not guaranteed or endorsed by the publisher.
